# Microbial synthesis of chalcogenide semiconductor nanoparticles: a review

**DOI:** 10.1111/1751-7915.12297

**Published:** 2015-06-25

**Authors:** Jaya Mary Jacob, Piet N. L. Lens, Raj Mohan Balakrishnan

**Affiliations:** ^1^ Department of Chemical EngineeringNational Institute of Technology Karnataka, SurathkalMangaloreKarnataka575 025India; ^2^ UNESCO‐IHE Institute for Water EducationDelftNetherlands

## Abstract

Chalcogenide semiconductor quantum dots are emerging as promising nanomaterials due to their size tunable optoelectronic properties. The commercial synthesis and their subsequent integration for practical uses have, however, been contorted largely due to the toxicity and cost issues associated with the present chemical synthesis protocols. Accordingly, there is an immediate need to develop alternative environment‐friendly synthesis procedures. Microbial factories hold immense potential to achieve this objective. Over the past few years, bacteria, fungi and yeasts have been experimented with as eco‐friendly and cost‐effective tools for the biosynthesis of semiconductor quantum dots. This review provides a detailed overview about the production of chalcogen‐based semiconductor quantum particles using the inherent microbial machinery.

## Introduction

The fabrication and utilization of matter at the nanoscale have attracted both academic and scientific interest since their inception. One such important class of nanomaterials that has allured the global scientific population is the semiconductor nanocrystals (NCs), also known as quantum particles, quantum dots (QDs) or quantum rods (QRs). Of particular relevance is the quantum particles composed of groups II–VI or III–V elements having unique electronic and optical properties resulting from quantum confinement effects (Li *et al*., 2007a, 2007b). These nano‐sized crystals with photoresponse in the near infrared region have opened an entirely new arena of research in the area of semiconductor nanocomposites (Syed and Ahmad, 2013) to better utilize their properties in expanses like cell labelling, cell tracking, DNA detection, *in vivo* imaging, biosensors, telecommunication, LEDs, lasers, photodetectors and photovoltaic devices (Jiang, 2012).

The remarkable optical perspectives of quantum particles have accredited them as ideal nanomaterials for ultrasensitive optical sensing for the selective detection of pesticides, organic compounds, biomolecules and a multitude of metals (Frasco and Chaniotakis, 2009). The conjecture regarding their efficient energy confinement and the subsequent optical properties was explained by a process called Förster (fluorescence) resonance energy transfer, wherein the light energy absorbed by the QDs (donor) is transferred to a nearby acceptor species, such as an organic fluorophore (acceptor) (Clapp *et al*., 2006). Following this approach, the fluorescence activation or quenching induced by the direct interaction between the analyte and the QDs surface, unmodified or functionalized with a given ligand, has supported the use of QDs as excellent sensing assemblies.

The early phase in nanotechnology evidenced numerous chemical methods for the synthesis of chalcogenide semiconductor NCs with precision in terms of size, shape and properties (Manna *et al*., 2000; 2003). In general, QDs can be synthesized through various routes, namely vapour phase and liquid phase deposition, or via colloidal synthesis (Baek *et al*., 2008). Vapour–liquid phase deposition methods involve the epitaxial growth of semiconductor crystalline films on suitable solid substrates using gaseous or liquid precursors. For instance, ZnO nanocrystals were synthesized by a vapour phase transport process where zinc acetate dehydrate vapours were deposited onto a silicon wafer substrate under high temperature and argon atmosphere (Lu *et al*., 2006). Conversely, colloidal syntheses require the reaction of appropriate starting materials, e.g. the reduction of metal ions or the decomposition of a single organometallic precursor in the presence of a surfactant or polymer that prevents the particles from growing or aggregating into larger sizes. Both synthesis approaches are commonly used, especially in the electronics industry, for coating materials with thin films, but have also been used to produce nanoparticles.

Although these synthesis methodologies have opened up new routes for preparing semiconductor NCs with tunable shape, size and optical properties (Lifshitz *et al*., 2003; Guo and Wang, 2011; Gutiérrez *et al*., 2012), the environmental aspects of these manufacturing processes are often not addressed (Thakkar *et al*., 2010). For instance, the chemical synthesis of cadmium selenide QDs imposes a high organic and inorganic pollutant load on air, water and soil. While sulfur, sodium, iron, oils and chloride ions are the dominant pollutants of water during the chemical synthesis of CdSe, oxides of sulfur, nitrogen and carbon are the major by‐products released into the air (Sengül and Thomas, 2009). Moreover, the chemical synthesis uses several organophosphorus solvents; their cost can account for up to 90% of the total production costs (Sirinakis *et al*., 2003).

The choice of the solvents is thus an important concern affecting the cumulative impact of the chemical synthesis routes. For example, in the trioctylphosphine oxide (TOPO) solvent, when the methyl radicals formed during the reaction combine with the oxygen in TOPO, ethers may form (Collins, 2005), which subsequently form complexes with un‐reacted selenium, thereby varying the reaction yield between 25% and 97%. This in turn affects the NC size, heating time, energy consumption and precursor requirement (Yu and Peng, 2002).

The development of eco‐friendly and green approaches for synthesizing these nanoclusters is thus required (Parikh *et al*., 2011; Ramanathan *et al*., 2011). Seeking lessons from nature's tools in assembling miniature functional materials in biological systems in elegant and ingenious ways, scientists have turned their focus to harness the biological factories to synthesize nanomaterials (Sastry *et al*., 2003). But the biosynthesis of chalcogen‐based semiconductor nanomaterials is relatively new and largely unfathomed. The sections below provide a detailed overview of the production of chalcogen‐based semiconductor quantum particles using the microbial machinery.

## QD materials

### Candidate materials and their properties

Candidate QD materials usually consist of elemental combinations of main groups II and VI, IV and VI, or III and V of the periodic table. Since the discovery of the first ever QDs in 1980, material scientists have focused on harnessing the potential of semiconductors like CdS, CdSe, ZnS, ZnSe, CdTe, InP, PbTe, GaAs and InAs (Ayee, 2008). An essential property of the ideal element for QD fabrication is that the material is a direct band gap semiconductor for which quantum confinement effects on size tuning are observable (Machol *et al*., 1994). The material possesses further a preferably narrow band gap with a high dielectric constant. The properties of some ideal QD elemental combinations are overviewed in Table 1.

**Table 1:** Biosynthesis of cadmium‐ and lead‐based chalcogenide

Semiconductor NC	Organism	Site of biosynthesis	Size and shape	Spectral properties	Other characteristics	Ref
Cadmium telluride (CdTe)	*Escherichia coli*	Extracellular	Monodisperse 2–3 nm, QDs	Fluorescence emission: 488–551 nm	Folic acid functionalized QDs used for bioimaging cervical cancer cell lines	Bao and colleagues (2010a)
	*Saccharomyces cerevisiae*	Extracellular	Monodisperse 2–3.6 nm, QDs	Fluorescence emission: 492 nm	Fluorescent, biocompatible QDs used in bioimaging	Bao and colleagues (2010b)
	*Fusarium oxysporum*	Extracellular	Polydisperse 15–20 nm, QDs	Fluorescence emission: 475 nm	Thermally stable with antibacterial properties	Syed and Ahmad (2013)
	*Lumbricus rubellus* earthworm	Intracellular	Monodisperse 2.33 + 0.59 nm, QDs	Fluorescence emission: 460, 520 nm	Crystalline water‐soluble luminescent nanoparticle; native and PEG‐capped QDs used in cellular imaging	Stürzenbaum and colleagues (2013)
Cadmium sulfide (CdS)	*Candida glabrata* and *Schizosaccharomyces pombe*	Intracellular	Monodisperse 20 Å, QDs	–	Peptide‐capped particles, short chelating peptides (γ‐Glu‐Cys)n‐Gly control the nucleation and growth	Dameron and colleagues (1989a, 1989b), Dameron and Winge (1990)
	*Klebsiella pneumonia*	Extracellular	Polydisperse > 5 nm	Absorbance maximum: 381, 424 nm	Band gap: 3.25, 2.9 eV, cysteine desulfhydrase assisted synthesis	Holmes and colleagues (1997)
	*Schizosaccharomyces pombe*	Intracellular	Monodisperse 1–1.5 nm, QDs	Absorbance maximum: 305 nm	Wurtzite (Cd_16_S_20_)‐type hexagonal lattice structure	Kowshik and collagues (2002a)
	*Escherichia coli*	Intracellular	Monodisperse 2–5 nm, QDs	–	Bacteria in stationary phase release thiol compounds to assist NC formation	Sweeney and colleagues (2004)
	*Fusarium oxysporum*	Extracellular	Polydisperse 5–20 nm, QDs	Absorption maximum: 450 nm	Sulfate‐reducing enzyme‐based process	Ahmad and colleagues (2002)
	*Rhodopseudomonas palustris*	Extracellular	Monodisperse 8 nm, QDs	Absorption maximum: 425 nm	Face centred cubic lattice crystallinity, cytoplasmic cysteine desulfhydrase enzyme	Bai and colleagues (2009)
	*Coriolus versicolor*	Extracellular	100–200 nm, Spherical	Fluorescence peak: 450 nm	Cd bioremediation and CdS synthesis in a continuous column mode	Sanghi and Verma (2009)
	*Brevibacterium casei*	Intracellular	Polydisperse 10–30 nm, QDs	Fluorescence peak: 430 nm	PHB encapsulated NCs with enhanced stability, reduced toxicity, used for bioimaging	Pandian and collagues (2011)
	*Escherichia coli*	Intracellular	Monodisperse 6 nm, QDs	Fluorescence emission: 445–510 nm.	Genetically engineered to introduce CdS‐binding peptide; water‐soluble biocompatible	Mi and colleagues (2011)
	*Lactobacillus sp*.	Extracellular	Monodisperse 4.93 nm, QDs	Maximum absorbance: 393 nm	Band gap energy: 2.52 eV; biosynthesis initiated by membrane bound oxidoreductases	Prasad and Jha (2010)
	*Saccharomyces cerevisiae*	Extracellular	Monodisperse 3.57 nm, QDs	Maximum absorbance: 369 nm	Band gap energy: 2.607 eV	
	*Enterobacteriaceae*	Intracellular	Polydisperse 5–200 nm, QDs	Maximum absorbance: 450 nm	Optimized conditions: pH: 9, temperature: 30°C, growth phase: stationary	Mousavi and colleagues (2012)
	*Serratia nematodiphila*	Extracellular	Monodisperse 12 nm, QDs	Maximum absorbance: 420 nm	Stable particles with antibacterial activity	Malarkodi and colleagues (2013)
	*Klebsiella pneumonia*	Extracellular	Polydisperse 10–25 nm, QDs	Maximum absorbance: 420 nm	QDs with antimicrobial activity	Bick and colleagues (2000)
Lead sulfide (PbS)	*Torulopsis* yeast	Intracellular	Monodisperse 2–5 nm, QDs	Absorption maximum: 330 nm	Band gap of 3.75 eV	Kowshik and colleagues (2002b)
Cadmium selenide (CdSe)	*Fusarium oxysporum*	Extracellular	Monodisperse 2–7 nm, QDs	–	–	Kumar and colleagues (2007)
Lead selenide (PbSe)	*Aspergillus terreus*	Extracellular	Polydisperse 20–50 nm diameter, QRs	Absorption maximum: 375, 872 nm	Band gap of 3.75 eV, weak quantum confinement	Jacob and colleagues (2014)

In a quantum particle, absorption of a photon larger than the band gap energy causes an electron to get excited to a state high in the conduction band. Due to the large degree of confinement, the generated electron and hole form a bound state called an exciton (a neutral bound pair of electron and hole), where the binding energy within the exciton cannot be overcome through the absorption photon energy. Unlike in the bulk semiconductors, enhanced Coulomb interaction in QDs results in much more tightly bound excitons that force them to interact with each other, resulting in shifts in the energy spectrum (Konstantatos and Sargent, 2013).

The optical properties undergo the greatest enhancement when the QD radius is smaller than the exciton Bohr radius, a_B_ of the bulk semiconductor:

(1) $$a_{B}=\frac{4\pi \varepsilon \left(\infty \right)h^{2}}{e^{2}}\left(\frac{1}{m_{e}}+\frac{1}{m_{h}}\right)$$

where ε(∞) is the optical frequency dielectric constant, and m_e_, _h_ are the electron and hole effective masses (Schmitt‐Rink *et al*., 1987).

**Figure 1:**
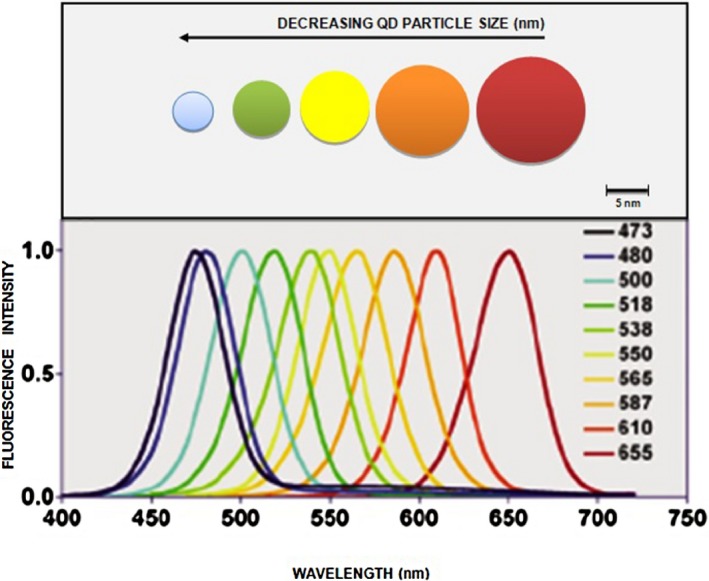
Size tunable fluorescence spectra of QDs (Tetyana and Yuri, 2011).

Based on Eq. (1), it can be explained that as the dimension of the bulk semiconductor is reduced, the density of states becomes concentrated, resulting in the narrowing of energy bands and eventually leading to discrete energy states. The oscillator strength of the strongly confined electron–hole pair inside a QD is predicted to be a factor of (a_B_/R)^3^ times larger than that of the bulk exciton (Harbold, 2005). This implies that QDs in the strong confinement limit have the potential for greatly enhanced optical properties and motivates the study of semiconductor QDs in this limit. Quantum confinement leads to altered emission lifetimes as well as altered luminescence quantum efficiency in quantum dots or rods (Overney and Sills, 2002). Additionally, quantum‐confined structures exhibit a shifted band edge that allows for the production of varied emission peak wavelengths as dictated by the size of the confinement (i.e. the size of the QD). Through strict dimensional control, QDs can be produced to emit narrow colour spectra that can be clearly distinct from one another at the full width half maximum of the peak emission with typical full‐width half maxima of only 25–30 nm. This size quantization effect with respect to the relative particle sizes is illustrated in Fig. 1.

### Cadmium‐based chalcogenide QDs

Cadmium sulfide (CdS) is often the nanoparticle semiconductor of first choice because of the ease of its fabrication: there is no high‐temperature, anaerobic chemistry involved and no molten solvents (Yang and Paul, 2003). Instead, CdS QD fabrication employs a reverse micelle aqueous solution in which the micelle's size dictates the size of the nanoparticle (Peng and Peng, 2001). Based on early semiconductor nanoparticle research on CdSe and ZnS (Kortan *et al*., 1990), Yang and Paul (2003) found that various reverse micelle methods can be used to produce both plain CdS QDs and CdS QDs doped with Mn. The difficulty with this method is that the resulting nanoparticle colloids are not monodisperse. Early CdSe QDs were produced using organometallic chemical techniques (Alivisatos, 1996). Thereafter, other groups, including Hines' and Bawendi's, synthesized colloidal CdSe QDs by other means as well (Danek *et al*., 1994). Furthermore, numerous efforts have been initiated to investigate new capping structures, such as dodecylamine and stearic acid, for CdSe (Hines and Guyot‐Sionnest, 1996; Peng *et al*., 1997; 2000; Peng and Peng, 2001).

Cadmium telluride (CdTe) QD production has generally been reported as a subset of CdSe preparation (Talapin *et al*., 2002), and hence little independent information is available about CdTe.

### Lead‐based chalcogenide QDs

In spite of technological advancements in the synthesis and characterization of II‐VI and III‐V semiconductors, it is noteworthy that the lower exciton Bohr radii in these materials do not allow access to the strong quantum confinement regime (Murray *et al*., 1993). In contrast, the IV‐VI semiconductors, and in particular the lead‐salt compounds, allow easy access to the strong confinement regime. Consider lead‐selenide (PbSe) and lead‐sulfide (PbS): the electron and hole masses are small and nearly identical (Madelung *et al*., 1982). This has the fortunate effect of leading to large Bohr radii, not only for the electron and the exciton, but for the hole as well (Murray *et al*., 1993). In PbSe, the electron, hole and exciton Bohr radii are 23, 24 and 47 nm respectively. These large radii allow strong confinements to be achieved in relatively large structures. Thus, QDs of IV‐VI materials may have properties reflecting all the benefits of strong quantum confinement, and with reduced influence from surface effects for the same level of confinement as QDs of II‐VI or III‐V materials, the surface‐to‐volume ratio can be quite low in IV‐VI materials. Strong confinement can then be achieved in relatively large QDs, with a smaller fraction of atoms on the surface. The properties of PbSe and PbS QDs are then far less likely to be influenced by surface effects like surface traps and defects (Wise, 2000). In II‐VI and III‐V semiconductors, the confinement induces coupling between the three (heavy‐hole, light‐hole and split‐off) valence bands. This further complicates the already dense ladder of hole states and leads to congested energy spectra (Ekimov *et al*., 1993).

The IV‐VI semiconductors have simple non‐degenerate conduction and valence bands. Additionally, their similarly small electron and hole masses lead to a large and nearly equal partitioning of the confinement energy between both charge carriers. Ultimately, these attributes result in sparse electron and hole states and simple energy spectra (Wise, 2000). Studies of extremely confined IV‐VI QDs have revealed that these materials have unique vibrational modes (Murray *et al*., 2001) that can exhibit extremely weak electron–phonon coupling (Kang and Wise, 1997). Further, these moieties have negligible exchange and Coulomb energies (Andreev and Lipovskii, 1999), and a temperature‐independent band gap (Allan and Delerue, 2004). Lead‐salt QDs are among the few materials that can provide size‐quantized electronic transitions at technologically important infrared wavelengths. These structures may find use in optoelectronic applications, as well as in biophysical applications such as fluorescence microscopy.

### Other Quantum Particle (QP) candidate materials

Zinc sulfide (ZnS) QDs and zinc selenide (ZnSe) QDs have each been synthesized by numerous groups around the world. Current research focuses on producing larger quantities, doping basic ZnS with manganese, lead (II) or cadmium, and varying the capping structures of the QDs (Yoffe, 2001). On the other hand, indium phosphide (InP) QDs demonstrate a large number of surface trapped states where electron‐hole non‐radiative recombination can occur. Further, spectrophotometric work on these InP QDs characterized them as absorbing in the visible spectrum towards the longer wavelength regions (Micic *et al*., 1995).

## Microbial QD synthesis mechanisms

Numerous biological resources have been exploited for bioproduction of Nanoparticles (NPs), including bacteria, fungi, algae, viruses, plants and plant extracts. Bacterial structures, such as the S‐layer, flagella and spores, are regarded as potent green nano‐factories for the production of NPs with exquisite sizes and shapes. Viruses are also fascinating nano‐biotemplates that can form NP arrays based on their inherent nanostructures, e.g. capsids (Narayanan and Sakthivel, 2010).

Because of their biotransformation and metal bioaccumulation ability, fungi and bacteria are receiving special focus for the biological generation of metallic nanoparticles (Sastry *et al*., 2003). While fungi offer a distinct advantage in nanoparticle synthesis owing to the ease in their scale‐up (e.g. using a thin solid substrate fermentation method), bacterial‐mediated synthesis in cell suspensions has also been reported for chalcogenide semiconductor nanoparticles. Given that microorganisms, especially fungi, are extremely efficient secretors of extracellular enzymes, it is possible to easily obtain large‐scale production of enzymes. Further advantages of using fungal‐mediated green approaches for synthesis of metallic nanoparticles include economic viability and ease in biomass handling (Ahmad *et al*., 2002). However, bacterial‐mediated nanoparticle synthesis confers advantages of directed evolution or genetic manipulation for the overexpression of specific enzymes identified in the synthesis of metallic nanoparticles. In this context, the economic viability and non‐toxic nature of the green routes for QD synthesis need to be highlighted, as these circumvent high temperature, pressure, energy or hazardous chemicals, and sophisticated operational requirements (Singh *et al*., 2010). For instance, while biosynthesis of CdSe QDs occurs at conditions akin to room temperature (Holmes *et al*., 1997), the chemical syntheses routes require higher process requirements, temperatures as high as 300°C and the presence of complexing agents like TOPO (Sengül and Thomas, 2009). As a result, the operational costs during scale‐up of chemical ‐mediated processes would increase, and the downstream pollutant load on the environment is expected to be high.

### Intracellular biosynthesis

Biosynthesis of semiconductor NCs was pioneered as early as 1989 in the yeasts *Candida glabrata* and *Schizosaccharomyces pombe*, cultured in the presence of cadmium salts (Dameron *et al*., 1989a, 1989b; Dameron and Winge, 1990). Short chelating peptides with the general structure (γ‐Glu‐Cys)n‐Gly were found to control the nucleation and growth of CdS crystallites to peptide‐capped intracellular particles of 20 Å diameter. These mechanistic insights into the biosynthesis of CdS affirm the findings (Dameron *et al*., 1989a, 1989b) that the yeast, upon exposure to cadmium salts, synthesizes metal chelating peptides (phytochelatin analogues). Upon addition of the metal, a Cd‐γ glutamyl complex is initially formed, and this is accompanied by an increase in the intracellular sulfide levels. These CdS complexes form CdS nanocrystallites that accumulate in the vacuoles.

Later, the intracellular biogenesis of CdS and PbS nanoparticles was initiated in *S. pombe* (Kowshik *et al*., 2002a) and *Torulopsis* (Kowshik *et al*., 2002b) strains respectively. Although the authors do not provide any mechanistic insights to the biosynthesis process, it is reported that the yeast initiated intracellular biosynthesis in the mid‐log phase of their growth. While enzymatic processes in sulfate‐reducing bacteria are relatively well understood and identified in the formation of biofilms of sphalerite (ZnS) and CdS, the intracellular synthesis of CdS in yeast occurs by a process involving sequestering of the Cd^2+^ ions by glutathione‐related peptides and a consequent production of CdS within the yeast cells (Dameron *et al*., 1989a, 1989b).

A shortcoming of the above‐discussed studies was that the metallic nanoparticles were synthesized ‘intracellularly’. Indeed, when the site of nanoparticle synthesis is intracellular, downstream processing becomes difficult and often defeats the purpose of developing a simple and cheap process (Kowshik *et al*., 2002a). The intracellular biosynthesis of CdS QDs in genetically engineered *Escherichia coli* (Mi *et al*., 2011) exemplifies the various recovery and purification steps generally followed in case of intracellular biosynthesis protocols. The *E. coli* cells were lysed using a hyper‐acoustic cell grinder, the lysed cell suspension was centrifuged, the re‐suspended cells were further freeze‐thawed at −70°C, and the QDs were purified using anion exchange resin columns. Pandian and colleagues (2011) also reported the microbial cell lysis using an ultrasonic disrupter for the recovery of CdS QDs from *Brevibacterium casei* cells prior to their characterization. Similar recovery and purification steps have been reported by other researchers for the intracellular biosynthesis of chalcogenide QPs (Mousavi *et al*., 2012), thus necessitating the need to explore alternate biosynthesis pathways that can surpass the subsequent treatments for the separation and purification of QPs and provide scalable routes for the large‐scale synthesis of QPs using microbial systems.

### Extracellular biosynthesis

**Figure 2:**
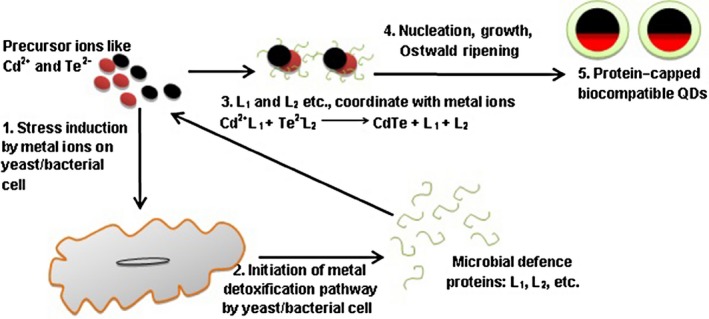
Microbial mechanism for the formation of biocompatible QDs.

To overcome the downstream processing steps of intracellular means of QD biosynthesis, one‐step scalable extracellular biogenesis of semiconductor nanocrystallites in microorganisms has been developed. In this regard, the extracellular biosynthesis of CdS NPs using the fungus *Fusarium oxysporum* in a facile enzymatic process has been reported (Ahmad *et al*., 2002). According to Bao and colleagues (2010a), a purely enzymatic process involving the release of specific enzymes such as reductases secreted by the microbe as a part of its defence mechanism could be responsible for the extracellular biosynthesis of QDs in solution. This pioneer effort opened up the exciting possibility of designing a rational biosynthesis strategy for nanomaterials of different chemical compositions. The synthesis process was rapid, scalable and could be carried out in conditions akin to working ambience. Bao and colleagues (2010a) have further detailed the mechanism of yeast‐mediated extracellular biosynthesis of CdTe QDs. These authors have speculated that the formation of protein‐capped CdTe QDs with uniform size involves an extracellular growth mechanism comprising of nucleation of metal ions with the yeast secreted proteins followed by Ostwald ripening. Upon simply mixing the Cd and Te precursors in the medium without yeast cells, only an amorphous Te colloid is obtained. The Te^2−^ and the Cd^2+^ ions in the reaction mixture when incubated with the yeast cells induce a specific defence mechanism generating proteins to coordinate with Cd and Te ions in an effort to detoxify the metal ions. In this biochemical process (Fig. 2), yeast‐secreted proteins coordinated with Cd^2+^ and Te^2−^ ions can eventually become incorporated as caps on the surface of the QDs forming a coating layer (Bao *et al*., 2010b).

According to the authors (Bao *et al*., 2010b), these caps not only improve the biocompatibility, but also ensure enhancement in fluorescent properties. The formation of microbial protein caps on the biogenic QDs can be considered as a deliberate leverage of these biosynthetic routes owing to the hydrophilicity of the QPs due to the surrounding peptide coat (Ghasemi *et al*., 2009), which further enables their application in biosensing and biolabelling (Bruchez *et al*., 1998; Chan and Nie, 1998). The microbial protein caps on the biogenic QDs also initiate surface modification on them, leading to a reduction in the local trap states and hence significantly increasing the efficiency of the excitonic emissions (Gao *et al*., 1998). Previously, Zhu and colleagues (2013) had described the enhanced photoluminescence in chemically synthesized Cd‐based QDs capped by thiol groups of thioglycolic acid.

The protein cap confinement also leads to a uniform size and good degree of dispersion without any aggregation. According to Bao and colleagues (2010b), the CdTe clusters (nuclei) subsequently grow in the medium following an Ostwald ripening process, resulting in the protein‐capped CdTe QDs with tailored size and excellent crystallinity. The CdTe QDs obtained by the extracellular growth approach can be easily isolated from the yeast cells by centrifugation, showing an obvious advantage over processes for the intracellular biosynthesis of CdS (Kang *et al*., 2008) and CdSe (Cui *et al*., 2009) QDs that require complicated procedures, including cell wash, disruption and fragment removal to obtain the isolated QDs.

Another important research effort for the extracellular biosynthesis of CdS nanoparticles in *Saccharomyces cerevisiae* was initiated by Prasad and Jha (2010). It is reported that fungal enzymes like oxidoreductases are activated by the yeast to quell the metal stress in solution. Such a stress‐generated response had earlier been suggested in the case of *Candida glabrata* cells exposed to cadmium ions in the form of excretion of the enzyme phytochelatin synthase and a protein HMT‐1, which effectively aborts the CdS NCs from cytosol. Once entered into the cytosol, the CdS might have triggered the family of oxygenases harboured in the endoplasmic reticulum, chiefly meant for cellular‐level detoxification through oxidation/oxygenation. The authors have also drawn a comparison of the efficiency of bacteria and fungi as candidate systems for biosynthesis. Based on their findings, the authors concluded better size quantization effects in yeast in comparison to bacterial‐mediated synthesis for the production of CdS nanoparticles (Prasad and Jha, 2010).

The involvement of 5′‐adenylylsulfate reductases in sulfate‐assimilating bacteria that could efficiently detoxify cadmium ions by the formation of extracellular CdS particles has also been detailed (Bick *et al*., 2000). Drawing inferences from the metal detoxification mechanisms in microorganisms, a comprehensive mechanistic insight into the biogenesis of CdS quantum particles in *Klebsiella pneumonia* was proposed (Malarkodi *et al*., 2013). According to the authors, initially the sulfate ions are taken up by the microbe from the nutrient broth that acts as extracellular environment and converted into adenosine phosphosulfate with the support of the enzyme ATP sulfurylase (Bick *et al*., 2000). The adenosine phosphosulfate is further phosphorylated to form 3′ phosphoadenosine phosphosulfate, which is subsequently reduced to sulfite ions with the aid of the enzyme phosphoadenosine phosphosulfate reductases (Lovley, 1993). The sulfite ions are further reduced to sulfide ions using sulfite reductases secreted as a part of the microbial metal detoxification system. The sulfide ions later couple with the inorganic metal ions (like cadmium) in the extracellular environment to form CdS nanoparticles (Bick *et al*., 2000; Auger *et al*., 2005).

Similar observations explaining the involvement of a 30 kDa protein in the extract of *Capsicum annuum* that could effectively reduce selenite to selenide ions through an enzymatic reaction was also reported (Li *et al*., 2007a, 2007b). In another study, it was reported that yeast cells incubated with Na_2_SeO_3_ and subsequently with CdCl_2_ resulted in the intracellular synthesis of CdSe QD and an increase in the glucan content of their cell walls, resulting in their enhanced mechanical strength (Luo *et al*., 2014). Genetically engineered *E. coli* strains harbouring plasmids containing phytochelatin synthase from *S. pombe* and γ‐glutamylcysteine synthetases are effective biofactories for the synthesis of CdS QDs (Kang *et al*., 2008). The important role of phytochelatin synthase in the synthesis of semiconductor quantum particles using histidine‐tagged phytochelatin synthase gene expressing *E. coli* cell lysates was also demonstrated (Liu *et al*., 2010). The phytochelatins were separated from the cell lysates by mixing with nickel resins based on nickel–hexa–histidine affinity interaction. The immobilized phytochelatin synthase converted the glutathione into the metal binding peptide phytochelatin, thus mediating the synthesis of phytochelatin‐capped CdS nanocrystals.

Regardless of the site of QP synthesis, it is noteworthy that the microbial sources for biosynthesis are largely unexplored and that a more comprehensive understanding regarding the mechanisms that initiate biosynthesis holds significance. According to Ramezani and colleagues (2010), the metabolic complexity of viable microorganisms complicates the identification of active microbial species in the nucleation and growth of NPs. In general, strategies such as enzymatic oxidation or reduction by the membrane bound, as well as cytosolic oxidoreductases (Ortiz *et al*., 1995), NADH‐dependant reductases and quinones (Prasad and Jha, 2010), activation of lyases (Bai *et al*., 2009) and an increase in the cellular pool of metal binding peptides (Krumov, 2009), have been developed and used by microorganisms for the biogenesis of NPs. Further studies on the biochemical and molecular mechanisms that mediate such processes and exploration of the enzymes involved may help improve our understanding of such processes to achieve the synthesis of smaller and monodisperse QDs using biomimetic means.

## Biogenesis of QDs

Although the constraints in the cultivation of microorganisms and the shape and size control of the microbiologically generated NPs have been reported as significant drawbacks of biomediated processes (Gericke and Pinches, 2006), several authors have overcome these limitations by optimizing the growth conditions for the microorganisms through adjustment of factors such as the pH, incubation time, temperature, metal salt concentration and the amount of biological inoculum, together with a variety of physical factors.

**Figure 3:**
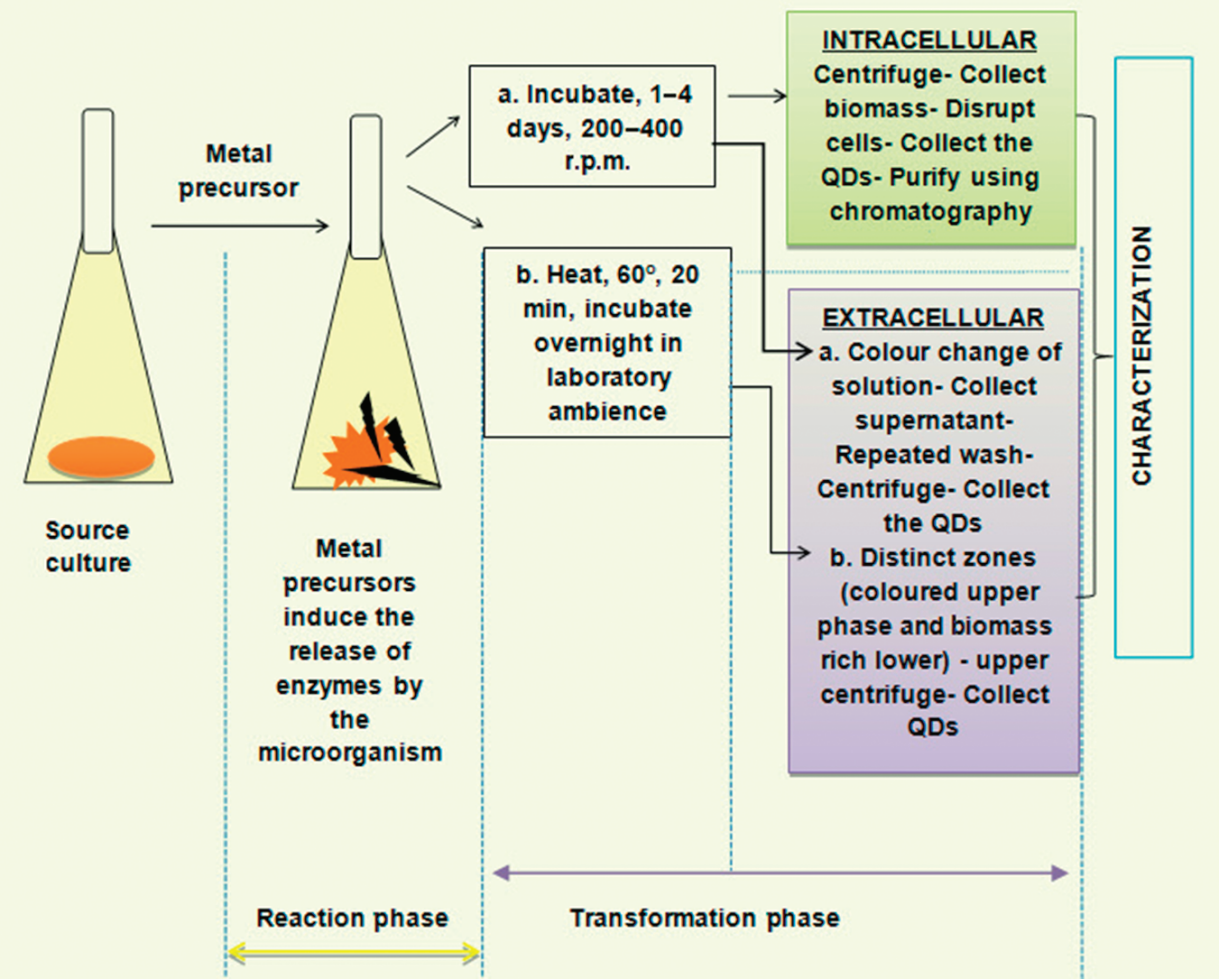
General scheme followed for biosynthesis of quantum particles by microorganisms.

**Figure 4:**
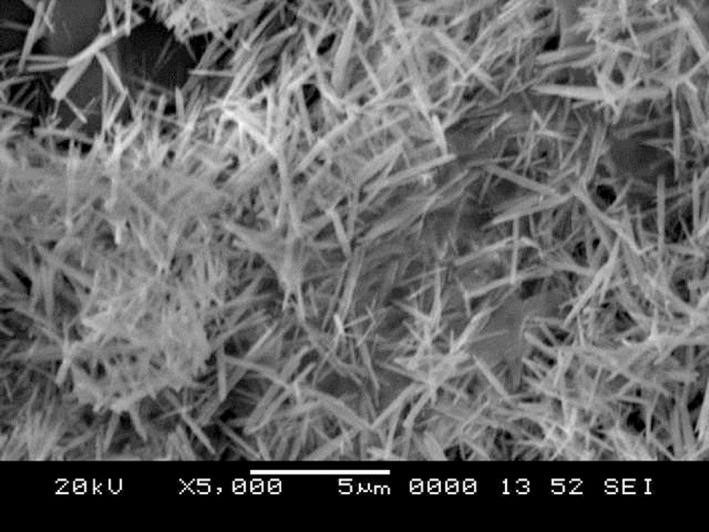
SEM image of the PbSe quantum rods biosynthesized in marine *A* *spergillus terreus*.

Table 1 summarizes the various efforts to biosynthesize cadmium and lead‐based chalcogenide semiconductor quantum particles. Both eukaryotic and prokaryotic organisms have been used in the biogenesis of quantum particles. Further, it is noteworthy that all reported procedures followed for biosynthesis trail to a similar scheme (Fig. 3), with the respective microorganisms being incubated with the metal salts for a given period over time to initiate a metal detoxification pathway in microbes, leading to the formation of the respective quantum particle. Figure 4 depicts an SEM image of PbSe QRs biosynthesized in marine *Aspergillus terreus*.

Williams and colleagues (1996) reported the process control for the microbial production of CdS nanocomposites departure from the traditional synthesis schemes for semiconductor quantum particles. These researchers were driven by the idea that CdS quantum semiconductor yield may be increased in *S. pombe* by maximizing the biomass yield under conditions where glucose repression is absent. To achieve this, they developed a feedback controlled fed‐batch system for the increased yield of biomass available for continuous CdS production. The degree of *S. pombe* glucose repression caused by the addition of cadmium was reduced so as to induce CdS QDs production. The study concluded that the glucose feed rate reduction followed by minimal glucose repression and ethanol production maximized the biomass yield and hence the QD yield. Further, the yeasts *S. pombe* and *Candida glabrata* were successfully cultivated in a fed‐batch process at cadmium levels up to 100 mg l^−1^ (Williams *et al*., 2002). *Schizosaccharomyces pombe* incorporated higher amounts of cadmium per gram of dry biomass compared with *C. glabrata*. The higher Cd uptake from *S. pombe* cells was correlated with the elevated glucose concentrations during the cultivation (Williams *et al*., 2002).

## Conclusion

Chalcogen‐based semiconductor QDs have initiated an application‐oriented research in nanotechnology. These NCs with excellent quantum confinement effects are mostly prepared by chemical means, which raise toxicity and cost‐related issues. Thus, there is a critical need in the field of nanotechnology to develop reliable and eco‐friendly protocols for the synthesis of QDs. The microbial community is relatively unexplored for their biosynthesis machinery for the production of chalcogenide QDs. Biological systems have the capacity to initiate the synthesis of these nanoclusters using the enzymes in their metabolic pathways at ambient temperature, pressure and neutral pH. Quantum dots can be synthesized both in bacteria and fungi, with fungal‐based systems having an advantage of easy handling and extracellular synthesis. Although bacterial synthesis has an advantage of involving directed evolution or genetic manipulation, fungi have a niche with respect to the cost factor on scale‐up and large‐scale synthesis. This area of nanotechnology offers future scope for exploring newer biosynthetic sources and the optimization of production conditions for the commercialization of QD to apply them in solar power harnessing and biosensing.

## Conflict of Interest

None declared.
